# The complete chloroplast genome sequence of *Oryza sativa aus*-type variety Nagina-22 (Poaceae)

**DOI:** 10.1080/23802359.2017.1407710

**Published:** 2017-11-25

**Authors:** Yeisoo Yu, Hyun-Oh Lee, Joong Hyoun Chin, Han Yong Park, Soo-Cheul Yoo

**Affiliations:** a Phyzen Genomics Institute, Seongnam, Korea;; b Graduate School of Integrated Bioindustry, Sejong University, Seoul, Korea;; c Department of Bioresources Engineering, College of Life Sciences, Sejong University, Seoul, Korea;; d Department of Plant Life & Environmental Science, HanKyong National University, Ansung, Korea

**Keywords:** *Oryza sativa*, *aus*-type, Nagina-22, N22, chloroplast genome, Poaceae

## Abstract

Rice (*Oryza sativa*) is the predominant staple food crop belonging to the Poaceae family. In this study, complete chloroplast genome sequence of *O. sativa aus*-type variety Nagina-22 was characterized through *de novo* assembly. The genome is a circular DNA molecule of 134,503 bp and has typical quadripartite structures including large single copy region (80,548 bp), small single copy region (12,347 bp), and a pair of inverted repeats (20,804 bp). A total of 120 genes were predicted in the genome, including 77 protein-coding genes, 8 open reading frame genes, 31 tRNA genes, and 4 rRNA genes. Phylogenetic analysis confirmed a close taxonomical relationship with *O. sativa* ssp. *Indica* species.

Rice (*Oryza sativa*) is the most important staple food crop belonging to the Poaceae family. Rice varieties are divided into two major subspecies, *Indica* and *Japonica*, which are further subdivided into five subpopulations, *indica, aus, temperate japonica, tropical japonica,* and *aromatic*. Of these, *indica* and *aus* belong to *Indica* subspecies (Garris et al. 2005; Zhao et al. 2010, 2011; Huang et al. 2012). Rice varieties of *aus* type have been used as valuable donor germplasms for tolerance genes, such as those for submergence-tolerance and phosphorus-deficiency tolerance (Xu et al. 2006; Gamuyao et al. 2012). An *aus*-type rice variety Nagina-22 (or N22) originated from India and shows tolerance against heat and drought (Satake and Yoshida 1978; Prasad et al. 2006; Jagadish et al. 2010; Vikram et al. 2010; Ye et al. 2012; Ye et al. 2015). Therefore, the variety is recognized as a good germplasm for development of new rice cultivar against global warming. In this study, chloroplast genome sequence of rice variety Nagina-22 was completed, which will enrich genetic resources and assist molecular breeding for *aus*-type variety.

Seeds of *O. sativa* cultivar Nagina-22 were originated from International Rice Research Institute (IRRI, GID 4537706 and IRIS 179-1440867) and cultivated by Hankyong National University (IRRI SMTA2017-0072) in Korea.

Genomic DNA was isolated from fresh leaves and subjected to constructing an Illumina paired-end (PE) library with 400 bp insert size, according to the manufacturer’s instruction. The library was sequenced using an Illumina Hiseq 4000 platform by Macrogen Co. (www.macrogen.com, Seoul, South Korea).

Chloroplast genome sequence was *de novo* assembled using about 977 Mb high quality PE reads and completed using manual correction and gap-filling, as described previously (Kim et al. 2015). The final complete chloroplast genome sequence was annotated using GeSeq (https://chlorobox.mpimp-golm.mpg.de/geseq-app.html) and manual curation by Artemis annotation tool (Rutherford et al. 2000) with NCBI BLASTN searches.

Complete chloroplast genome sequence (GenBank Accession no. MG252500) of *O. sativa* variety Nagina-22 is a circular DNA molecule of 134,503 bp, which has typical quadripartite structures consisting of large single copy (LSC) region of 80,548 bp, small single copy (SSC) region of 12,347 bp, and a pair of inverted repeats (IRa and IRb) of 20,804 bp. A total of 120 genes were predicted in the genome, including 77 protein-coding genes, 8 open reading frame genes, 31 tRNA genes, and 4 rRNA genes. Overall GC content of the genome is 39%.

Phylogenetic analysis was performed based on multiple alignments of chloroplast genomes of 16 *Oryza* species. The phylogenetic tree showed that Nagina-22 was grouped much closer with *O. sativa* ssp. *Indica* species than ssp. *Japonica* species (Figure 1), which was similar to phylogenetic relationship based on nuclear genome SNPs (McNally et al. 2009).

**Figure 1.**
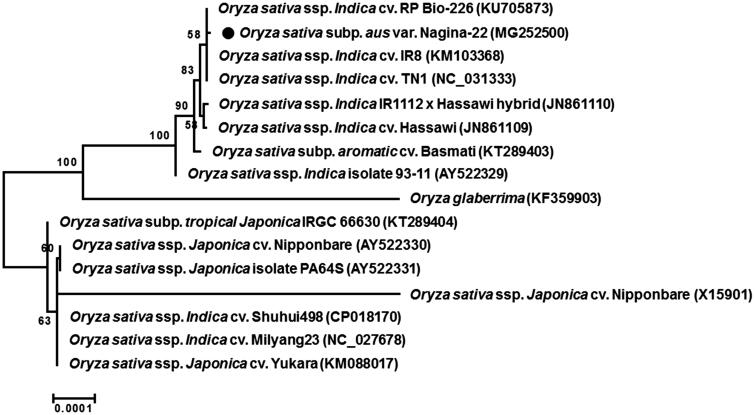
Phylogenetic tree of *O. sativa* variety Nagina-22 with related taxa. Whole chloroplast genome sequences were aligned using MAFFT (http://mafft.cbrc.jp/alignment/server/index.html) and used to generate maximum-likelihood phylogenetic tree by MEGA 6.0 (Tamura et al. 2013). The numbers in the nodes indicated the bootstrap support values (>50%) from 1000 replicates. Scale bar represents the number of nucleotide substitution per site. GenBank accession numbers of used chloroplast genome sequences are in parentheses. Abbreviation ssp., cv. and subsp. indicate subspecies, cultivar, and subpopulation, respectively.
